# Case Report: Successful treatment of steroid-refractory severe immunotherapy-induced pneumonitis with equine antithymocyte globulin

**DOI:** 10.3389/fonc.2026.1801207

**Published:** 2026-03-19

**Authors:** Thi Thao Vi Luong, Lindon Lin, John Coutsouvelis, Eli Dabscheck, Steven Ivulich, Hayley Burridge, Dominic Keating, Mark Shackleton, Andrew Haydon, Miles C. Andrews

**Affiliations:** 1 Department of Medical Oncology, Alfred Health, Melbourne, VIC, Australia; 2 Department of Pharmacy, Alfred Health, Melbourne, VIC, Australia; 3 Centre for Medicine Use and Safety, Monash University, Parkville, VIC, Australia; 4 Department of Respiratory Medicine, Alfred Health, Melbourne, VIC, Australia; 5 Department of Cancer Medicine, School of Translational Medicine, Monash University, Melbourne, VIC, Australia

**Keywords:** antithymocyte globulin, ATGAM, corticosteroid-refractory pneumonitis, immune checkpoint inhibitors, immunotherapy-induced pneumonitis

## Abstract

Immune checkpoint inhibitors (ICI) have revolutionized melanoma treatment but are associated with autoimmune toxicities. Immunotherapy-induced pneumonitis (IIP) is a potentially fatal immune-related adverse event. Current management of IIP involves corticosteroids, mycophenolate mofetil (MMF), intravenous immunoglobulin, or infliximab for severe cases. Limited data exist for corticosteroid-refractory pneumonitis. This case report is the first to describe successful treatment of refractory grade 4 IIP with equine antithymocyte globulin (eATG) after failure of corticosteroids and MMF. A 50-year-old woman with recurrent unresectable melanoma in the right ankle developed grade 4 IIP after receiving two cycles of ipilimumab and nivolumab. Despite intravenous high-dose corticosteroid and MMF, her clinical condition continued to rapidly deteriorate. eATG was administered due to its rapid onset of T lymphocyte depletion and its use in immunotherapy-induced myocarditis. An 8-day course was delivered with dose adjustment to achieve therapeutic CD2^+^/CD3^+^ lymphocyte depletion. Clinical and radiological improvement was demonstrated, with successful weaning of oxygen and corticosteroids. At 12 months, the patient remained well from a respiratory standpoint, with no recurrence of melanoma. This case highlights the potential for eATG, with therapeutic CD2^+^/CD3^+^ T lymphocyte count monitoring, to address an unmet therapeutic need in patients with refractory ICI-induced IIP.

## Background

Immune checkpoint inhibitors (ICI) have revolutionized the treatment for many malignancies. Combination immune checkpoint inhibitor (CICI) with ipilimumab (a cytotoxic T-lymphocyte-associated antigen-4 [CTLA-4] inhibitor) and nivolumab (a programmed cell death-1 [PD-1] inhibitor) has improved survival in melanoma (1). The rates and severity of immune-related adverse events (irAEs) are higher for CICI than for single-agent anti-PD-1 (e.g., grade ≥ 3 irAE ~ 55% for CICI *vs*. ~ 15% for anti-PD-1 monotherapy), leading to long-term morbidity and mortality (1, 2). A meta-analysis of 20 trials comprising 4,496 patients reported an incidence of immunotherapy-induced pneumonitis (IIP) ranging from 2.7% for anti-PD-1 monotherapy to 6.6% for CICI (3). Recent real-world studies suggest that the incidence of IIP might be underestimated, with numbers likely to increase as ICI therapy is used more routinely (4). While most cases of low-grade IIP resolve with corticosteroids, patients with refractory IIP experience up to 40%–60% mortality, with little robust evidence to support specific second- or third-line therapies (5). Case reports and small retrospective series describe the use of various agents, including infliximab, mycophenolate mofetil (MMF), intravenous immunoglobulins (IVIg), and interleukin (IL)-6 inhibitors (5, 6).

Equine antithymocyte globulin (eATG) is a polyclonal antibody against human T cells derived from horse thymus lymphoid cells, with a half-life of 2 to 3 days. Its immunosuppressive effect is mediated via rapid T-cell depletion, which begins 24 to 48 h after administration and can last weeks to months. Its use is currently indicated in acute cellular rejection of solid organ transplants and immune-mediated myocarditis (7). According to local lung transplant protocol, close monitoring of CD2^+^ and CD3^+^ lymphocyte counts is required to guide dosing and mitigate complications of prolonged or excessive immunosuppression; levels of 50–150 cells/μL are considered optimal for the recommended 5-day course of eATG. Use of eATG (ATGAM^®^, Pfizer Australia, Sydney, New South Wales, Australia) has not previously been reported in refractory IIP. We describe the successful treatment of a life-threatening IIP with eATG in a patient following CICI therapy.

## Case presentation

**Figure 1:**
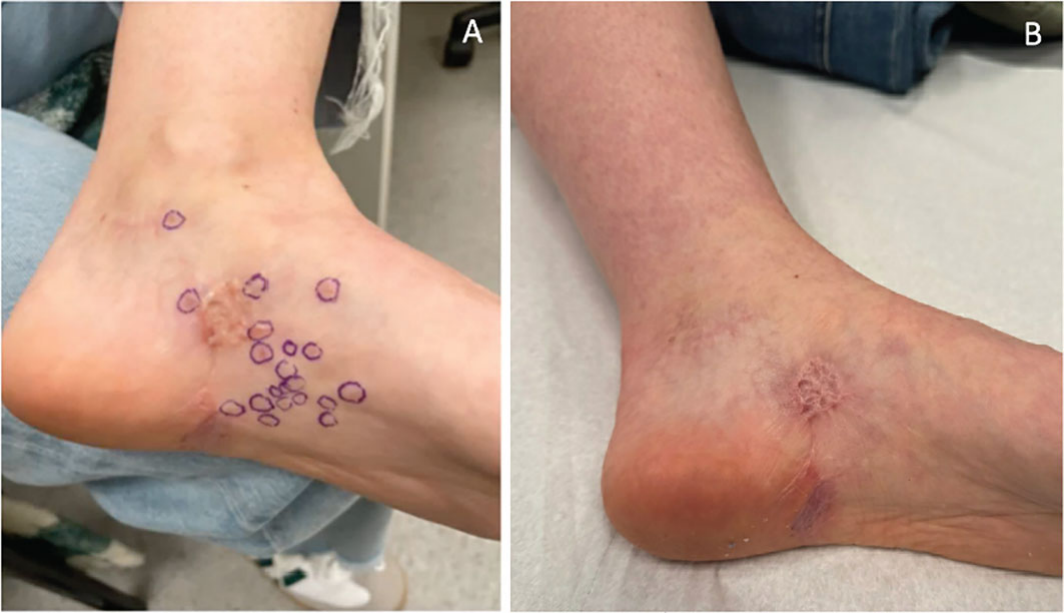
Clinical images of melanoma dermal metastases centered around the left ankle **(A)** prior to combination immune checkpoint inhibitor therapy and **(B)** after two cycles of CICI and 6 months after resolution of immunotherapy-induced pneumonitis.

**Figure 2:**
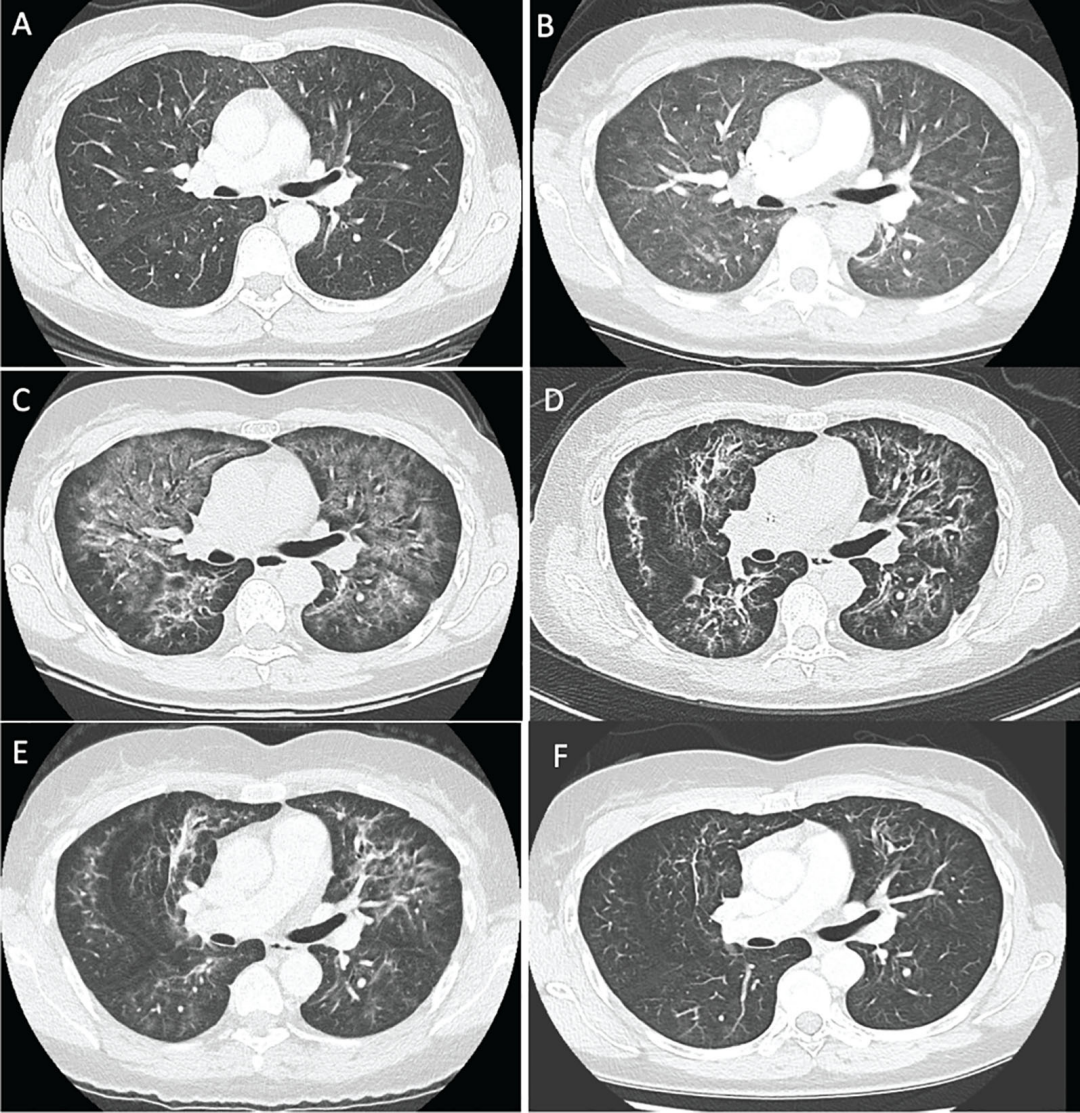
CT chest appearances at **(A)** baseline prior to immunosuppression, **(B)** following 2 weeks of oral corticosteroid therapy, **(C)** after intravenous methylprednisolone and mycophenolate, **(D)** after eATG, **(E)** 6 weeks after commencing eATG, and **(F)** at 9 months.

A 50-year-old woman underwent complete resection followed by 12 months of adjuvant nivolumab for stage IIIC *BRAF* V600E mutant melanoma of the left foot 3 years earlier. She subsequently developed multiple dermal recurrences around her left ankle, requiring several excisions over a 2.5-year period. One month prior to presentation, she developed new in-transit metastases on the foot, ankle, and proximal leg that were deemed unresectable (**Figure 1A**). An FDG-PET scan showed no evidence of distant metastatic disease, and CICI was commenced. After two cycles, she developed a dry cough and shortness of breath and was diagnosed with IIP. Computed tomography (CT) scan of the chest showed diffuse bilateral centrilobular ground-glass opacities (**Figure 2A**). Alternative diagnoses such as infection and cardiac failure were ruled out after extensive septic workup, including blood and sputum cultures, multiplex respiratory viral polymerase chain reaction (PCR), cardiac enzymes (troponin, creatine kinase), and transthoracic echocardiogram. Induction cycle three of CICI was withheld, and the patient was commenced on oral corticosteroids at 1 mg/kg with trimethoprim/sulfamethoxazole (800/160 mg) three times weekly for *Pneumocystis jirovecii* pneumonia (PJP) prophylaxis. After 2 weeks, symptoms progressed from grade 2 to grade 3 pneumonitis, with worsening tachypnea, hypoxia, and dependence on supplemental oxygen (**Figure 2B**).

Intravenous (IV) methylprednisolone was administered initially at 1 mg/kg for 3 days, then escalated to 200 mg/day for 3 days without any significant clinical improvement. MMF at 500 mg twice daily (BD) orally was added and uptitrated to 1,000 mg BD, but without improvement after 10 days. CT chest showed worsening of the diffuse bilateral peri-bronchovascular ground-glass infiltrates involving all lobes, with interval coarsening and mild to moderate traction bronchiectasis (**Figure 2C**). The extended viral panel, AFB smear for tuberculosis, PJP PCR, *Nocardia* species, *Aspergillus* DNA PCR, and galactomannan were all negative on bronchoscopy. Fungal elements were present in the bronchoalveolar lavage (BAL), with branching septate fungal hyphae suggestive of *Aspergillus*; however, fungal culture was negative, and invasive disease was considered unlikely following review by the Infectious Disease service. The BAL smear showed mucus with alveolar macrophages and small numbers of inflammatory cells, without organisms, malignant cells, or viral inclusions.

**Figure 3:**
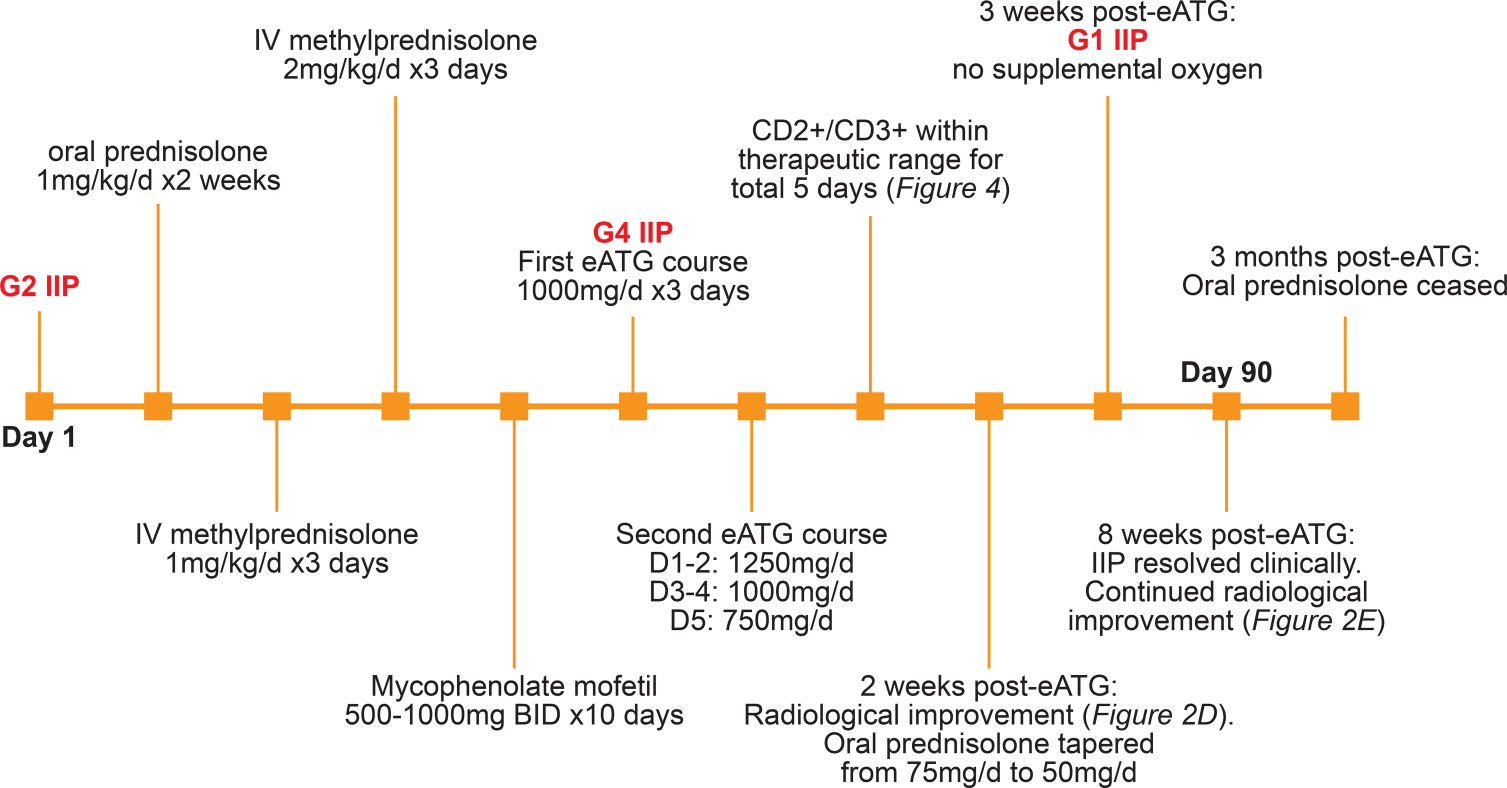
Timeline for IIP and response to treatment.

**Figure 4:**
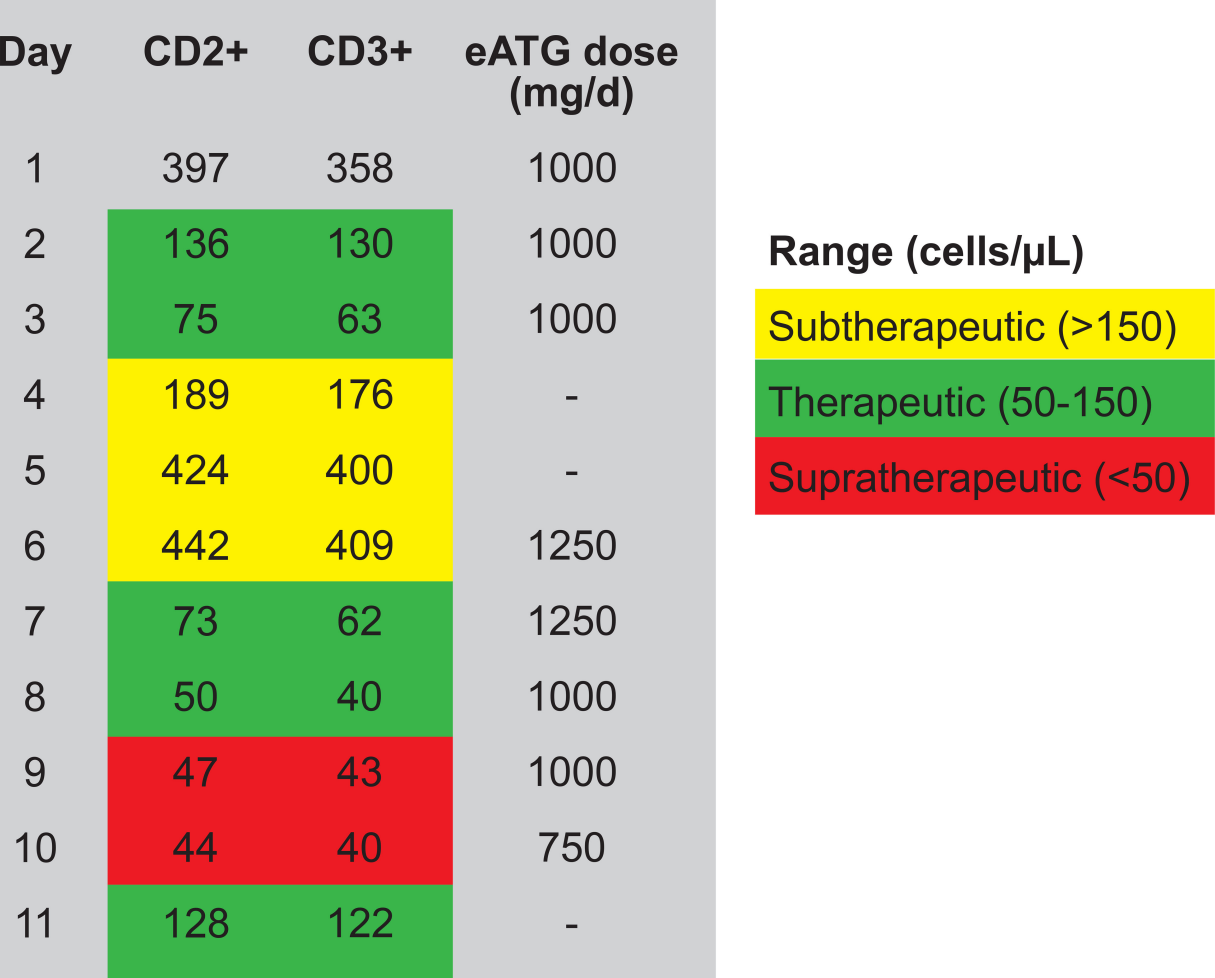
CD2^+^/CD3^+^ therapeutic level correlation with eATG dose.

Subsequent progression to grade 4 IIP led to the decision to use eATG as the next therapy based on a presumed fundamental T-cell-driven toxicity and the imperative to halt the disease process rapidly. Routine screening prior to eATG administration included hepatitis B/C, tuberculosis, cytomegalovirus, Epstein–Barr virus, and herpes simplex virus. Prophylactic valganciclovir at 450 mg BD, with premedication of methylprednisolone at 100 mg IV, was administered prior to eATG. Due to the presence of fungal elements, posaconazole at 300 mg daily was started prophylactically, targeting blood levels > 0.7mg/L. eATG was administered daily, targeting CD2^+^ and CD3^+^ levels between 50 and 150 cells/μL, with a maximum of 300 cells/μL. CD2^+^ and CD3^+^ levels were measured the morning after eATG administration to guide dose adjustments, and the drug was administered in the afternoon. Treatment was initiated with eATG at 1,000 mg daily for 3 days. CD2^+^ and CD3^+^ levels remained < 150 on days 2 and 3 but rose to > 150 on day 4 and > 300 on day 5. This correlated clinically with the patient’s unsustained response. A second course of eATG was therefore administered over 5 days, starting at a dose of 1,250 mg daily. CD2^+^ and CD3^+^ levels were within the target range for the first 2 days but fell below 50 on the third day; hence, the eATG dose was reduced to 1,000 mg daily on days 3 and 4. On day 5, the dose was further reduced to 750 mg daily as CD2^+^/CD3^+^ levels remained at a supratherapeutic level (i.e. too low) (**Figure 3**). The patient received a total 8-day course of eATG, 5 days of which had CD2^+^/CD3^+^ levels within the therapeutic target range (**Figure 4**).

The patient improved clinically and radiologically (**Figure 2D**) and was discharged 1 week after completing eATG on oral prednisolone 50 mg daily. This dose was subsequently tapered by 5 mg each week. By week 3, the hypoxia had resolved, and oxygen supplementation was weaned. Eight weeks post-eATG, CT of the chest continued to show improvement (**Figure 2E**). Clinical resolution of the in-transit disease at the left ankle was observed at this time (**Figure 1B**). Restaging FDG-PET/CT scan at 9 months showed ongoing improvement of interstitial changes with no evidence of locoregional or metastatic melanoma (**Figure 2F**), a response that was maintained at 12-month surveillance.

## Discussion

The pathogenesis of IIP remains poorly understood, although CD8^+^ T-cell activation and proliferation are likely to be involved (8). While CD8^+^ lymphocytosis is commonly found in studies of IIP, various other lymphocyte subsets, inverted CD4/CD8 ratios, and the release of interferon-gamma, IL-6, IL-17A, and IL-35 have all been implicated in the mechanism of IIP or the risk of IIP relapse (9).

It is essential to identify risk factors and predictive biomarkers for refractory IIP. The median time to onset of pneumonitis is shorter in patients developing severe IIP than in those developing milder disease (10). Increasing age, male sex, smoking history, baseline pulmonary function test abnormalities, higher baseline CD4^+^ lymphocyte count, previous lung disease, prior thoracic radiation, tumor histology type, and receipt of CICI have each been identified as risk factors for IIP (10, 11). Lower absolute baseline lymphocyte, lower red cell distribution width (RDW), and high eosinophil counts and albumin are associated with more severe IIP (11, 12). Other than receiving CICI, our case lacked clear risk factors for the development of IIP.

Many immunosuppressive agents have been trialed in steroid-refractory irAE (i.e. those with no or inadequate initial response), with evidence mainly extrapolated from case reports and small retrospective studies. MMF was successfully used as a steroid-sparing agent in two reported cases of steroid-resistant IIP (13). A study describing 26 steroid-resistant (i.e. those with loss of initial response) and refractory IIP patients demonstrated that steroid-refractory disease has a shorter response duration to immunomodulators (25% *vs*. 50%) and a lower 90-day survival (25% *vs*. 71%) compared to steroid-resistant cases (6). In another retrospective series of 65 IIP patients, 10 of 12 steroid-refractory patients (83%) ultimately died from refractory pneumonitis, infection, or progression of their cancers. In this study, none of the five steroid-refractory patients treated with infliximab ± IVIg survived. Two of seven (29%) patients treated with IVIg alone achieved improvement of symptoms; however, their baseline symptom severity may have differed, and long-term trends in steroid weaning or IIP recurrence were not reported (14). The high mortality rate observed with infliximab in IIP raises questions about the uncertain role and efficacy of this medicine in this population. IL-6 blockade (e.g., tocilizumab) has an established role in the management of cytokine release syndrome (CRS), including that resulting from T-cell engager therapies such as tebentafusp. Tocilizumab showed a 79% clinical improvement rate in a study of 87 patients with steroid-refractory irAEs; outcomes in the subset with grades 3–4 IIP (35%) were not specifically reported (15). Limited data are available regarding other agents, such as cyclosporine and cyclophosphamide, in IIP (5).

In this case, rapid clinical deterioration despite high-dose corticosteroid and MMF motivated the choice of eATG because of its potential for rapid T-cell depletion in a setting felt likely to be T-cell driven and without clinical features of a cytokine-driven phenomenon such as CRS. eATG has been successfully used in immune-mediated myocarditis, including a case in which prominent CD3^+^ and CD8^+^ lymphocyte infiltration was observed in endomyocardial biopsies, suggesting a similar T-cell-mediated basis of end-organ damage (7, 16).

The initial course of eATG did not achieve the target CD2^+^ and CD3^+^ levels, warranting another 5-day course with tighter dose titration, which led to the resolution of IIP. While potent immunosuppression was imperative to mitigate the high risk of mortality and long-term morbidity in this case, competing risks such as life-threatening infection or melanoma recurrence were present. However, rechallenge with immunotherapy is not a viable option in ≥ grade 3 IIP due to up to 50% risk of flares (17). Just as the evidence base to guide pharmacotherapy of steroid-refractory severe irAEs is limited, whether aggressive immunosuppression needed to treat severe irAEs (as opposed to baseline immunosuppression for other indications) meaningfully increases the risk of infections is unknown and of lesser immediate clinical relevance in the setting of fulminant irAE. Nevertheless, a comprehensive approach that includes adequate viral, fungal, and bacterial prophylaxis against opportunistic infection, while monitoring closely for CD2^+^ and CD3^+^ levels, is paramount to guide therapeutic dosing, mitigate complications, and achieve an adequate response. Notably, our patient demonstrated an ongoing complete response of their melanoma despite aggressive immunosuppression, without any major infectious complications.

## Conclusion

We report a case of refractory IIP treated successfully with eATG titrated to CD2^+^ and CD3^+^ lymphocyte counts, with sustained clinical improvement at 12 months. Larger prospective studies are needed to confirm its long-term efficacy and immunosuppressive impact on tumor control. eATG represents a new therapeutic option for severe refractory immune-related pneumonitis where conventional therapy with high-dose corticosteroid and MMF has been ineffective.

## Data Availability

The original contributions presented in the study are included in the article/Supplementary Material. Further inquiries can be directed to the corresponding author.
